# Socioeconomic Factors Associated With the Risk and Prevalence of Dental Caries and Dental Treatment Trends in Children: A Cross-Sectional Analysis of National Survey of Children's Health (NSCH) Data, 2016-2019

**DOI:** 10.7759/cureus.19184

**Published:** 2021-11-01

**Authors:** Deepa Vasireddy, Thevasha Sathiyakumar, Sumona Mondal, Shantanu Sur

**Affiliations:** 1 Pediatrics, Pediatric Group of Acadiana, Lafayette, USA; 2 Mathematics, Clarkson University, Potsdam , USA; 3 Mathematics, Clarkson University, Potsdam, USA; 4 Biology, Clarkson University, Potsdam, USA

**Keywords:** fluoride varnish, odds ratio, prevalence study, socioeconomic factors, healthcare insurance, household income, medical home, tooth decay, dental caries

## Abstract

Introduction

In the United States of America, early childhood caries (ECC) is the most common chronic childhood disease of early onset, with dental caries being the most prevalent chronic disease among children aged 6-19 years. Children without an established medical home, from low-income households, and who are uninsured have historically shown to be prone to dental caries and attribute to higher health care costs. Early recognition of these risk factors by a pediatrician helps prevent the development of medical and psychosocial complications in the child.

Methods

The cross-sectional data of the prevalence of dental caries and dental treatment trends in children and three socioeconomic risk factors, namely establishment of a medical home, household income, and child’s health insurance, were accessed from the National Survey of Children's Health (NSCH) for the years 2016-2019. The association of the risk factors with the prevalence of dental caries and with the prevalence of dental treatment were analyzed using two-sample proportion tests and chi-square (χ^2^) tests for dichotomous categorical variables and non-dichotomous categorical variables, respectively. Standardized residuals were calculated and analyzed as well. Furthermore, the odds ratios were calculated and utilized to quantify the influence of each category on the highly associated category with having teeth decay and not receiving dental treatment under each socioeconomic risk factor.

Results

The results of this study revealed that the three socioeconomic factors considered have statistically significant associations with tooth decay and dental treatment. The prevalence and associative risk of tooth decay and untreated caries were the highest in the children without a medical home. Additionally, the odds of having tooth decay was >50% higher for the children from the lowest household income category (0-99% federal poverty level [FPL]) compared to those from the high household income categories (200-399% FPL and >400% FPL). Public insurance coverage was associated with the highest prevalence of dental caries and not receiving fluoride treatment. Furthermore, the likelihood of not availing dental treatment is nearly two times or more higher for the uninsured children than children having public insurance, or private insurance, or a combination of both.

Conclusion

Our study findings reveal that children belonging to certain socioeconomic risk categories are at a higher risk of developing dental caries and not receiving dental treatment. As a consequence, the study implies that increased support and expansion of public health insurance will benefit oral health care for the children. Pediatricians play an integral part in developing a medical home for the child by providing preventative dental care and establishing continued care through dental referrals.

## Introduction

In the United States of America (USA), early childhood caries (ECC) is the most common chronic childhood disease of early onset, with dental caries being the most prevalent chronic disease among children aged 6-19 years [1,2]. Children represent around 33% of the population living in poverty, which is strongly correlated with childhood caries [3]. According to the United States Census Bureau’s 2018 report, 4.3 million children did not have health insurance coverage in 2018, which was a 0.6% increase from the year prior to it. This decline in the public insurance coverage for children is attributed to the decline in Medicaid and Children’s Health Insurance Program (CHIP) coverage rates [4]. Children from low-income families and in the age group of 5-19 years were found to be twice as likely to have cavities in their teeth compared to children from higher income households [5]. According to a study conducted on the 2007-2008 National Health and Nutrition Examination Survey (NHANES) data, higher socioeconomic status was associated with a lower prevalence rate of untreated dental caries [6]. Subsequent analysis of NHANES data from the years 2015 to 2016 showed the prevalence of both total and untreated caries to decrease with an increase in the family income level [7].

Early intervention of dental caries is highly favored as untreated cases can lead to complications increasing patient suffering and often causing a considerable amount of adverse psychosocial impact. Moreover, complex procedures that are frequently needed to treat advanced caries can only be performed by a small group of specialists, significantly restricting the treatment availability and increasing the health care cost. In one study, it was found that the rate of tooth decay was five times higher in children below the poverty line than those 300% or more above it [8]. Dental caries in children when left untreated can lead to infection, pain with secondary chewing difficulty, sleep disturbances, poor speech articulation, and lowered self-esteem, all of which could potentially lead to decreased quality of life. Pain precludes the child from eating, thus affecting their overall nutrition and eventually impacting the growth and development [1,9-13]. Children with discolored, missing, or damaged teeth often become conscious about their appearances, which makes them vulnerable to psychosocial impairment due to hesitancy to interact with others [13].

Management of caries typically involves an oral and radiographic examination followed by dental fillings or the placement of a stainless steel crown on the affected tooth or even both. According to one study conducted on 322 Alaskan Native children, the total estimated average annual costs of treating dental caries and full mouth dental restorations were $258,000 and $1.5 million, respectively [14]. It has been found that the Medicaid program alone in the USA pays between $100 million and $400 million annually to treat ECC [15]. Establishment of a dental home for the child is considered to be a key strategy in preventing the ECC. A dental home helps establish a coordinated family-centered access to preventative and interventional dental services. Pediatricians play a key role in getting a dental home established, which is recommended to be done as soon as the first tooth erupts [16].

Given the disease burden of dental caries, we wanted to analyze the socioeconomic factors that make a child susceptible to the development of the disease so that the primary care physician can identify and address the need for a dental care home for the child early on and help the family find the resources to have one established.

## Materials and methods

Data sources

The National Survey of Children's Health (NSCH) is a nationwide survey, which is designed by the Centers for Disease Control and Prevention's National Center for Health Statistics and sponsored by the Mental and Child Health Bureau of the Health Resources and Services Administration [17]. The survey is cross-sectional in nature, conducted annually, and utilizes a stratified sampling design. A paper survey instrument and a web-based survey instrument are used for data collection. The survey first identifies households with children from a national sample of addresses followed by randomly selecting one child from each eligible household. The selected child was then subjected to a more detailed topical questionnaire. The processed survey data are publicly available through the NSCH website. In this study, we used data for the years 2016-2019, which included 50,212, 30,530, 21,599, and 29,433 completed surveys for children during the years 2016, 2017, 2018, and 2019, respectively.

Study measures

This study focuses on two NSCH survey questions regarding dental caries and dental treatment among children aged 0-17 years. The first question inquires the presence of any decayed teeth or cavities with two response options: “yes (TD)” and “no (NTD)”. The second question inquires about the status of preventive dental services received by the child with three response options: “received fluoride treatment (RF)”, “received preventive dental care, but not fluoride treatment (RNF)”, and “did not receive preventive dental care (NR)”. The study analyzed the association of dental caries and treatment with three socioeconomic variables, also collected in the survey, as provided in Table 1.

**Table 1:** Socioeconomic variables used in the study FPL, federal poverty level

Study variable	Study categories used
Establishment of a medical home for the child	1. Care was met by medical home criteria
	2. Care not met
Household income	1. 0-99% FPL
	2. 100-199 % FPL
	3. 200-399% FPL
	4. >400% FPL
Child’s health insurance	1. Public
	2. Private
	3. Public and private
	4. Uninsured

Among these variables, the variable household income was categorized according to the federal poverty level (FPL), which is a standard measure provided by the U.S. Department of Health and Human Services.

Statistical analysis

Two statistical tests were used to analyze the associations between the socioeconomic variables and the prevalence of dental caries or dental fluoride treatment. The Z-proportion test was used to analyze the dichotomous variable “medical home criteria,” and the chi-square (χ^2^) tests of independence were used to analyze non-dichotomous variables “household income” and “insurance,” respectively [18]. The null hypothesis of the χ^2^ test states that there is no association between the categorical variables in the population and that they are independent. The larger the calculated values of χ^2^ test statistics, the more the contradictions to the null hypothesis. Similarly, smaller p-values (<0.05) are contradictions to the null hypothesis, where p-values are depicted as the null probability that χ^2^ is at least as large as the observed value. When the test identifies an association of the socioeconomic factors with the prevalence of dental caries as well as the dental fluoride treatment, standardized residuals, which often describe a cell-by-cell comparison, are used to estimate the strength of the association among each categorical combination as the next step [19,20]. The larger the positive standardized residuals of the cell, the higher the count of the categorical combination associated with that particular cell. On the other hand, the larger the negative standardized residuals, the fewer the count of the categorical combination associated with the particular cell than what the hypothesis of independence predicts [19]. Thus, we can infer strong positive and negative associations between the categories of each cell if larger positive and negative standardized residuals are obtained.

Once the test statistics for χ^2^ tests were found to be significant, standardized residuals were calculated to identify specific cells that were making the highest contribution to the test results. The association of socioeconomic risk variables with the prevalence of dental caries and dental treatment was further estimated by calculating the odds ratios (ORs) after dichotomizing each variable. Confidence interval (CI) constructed for an OR gave an expected range for the true OR for the population to fall within. The CIs that include 1 are not statistically significant. Higher values of OR imply increased odds of having the teeth decay or not having dental treatment to associate with the exposure of respective categories of socioeconomic risk variables.

## Results

Table 2 shows estimated prevalence of dental caries among children in each category of socioeconomic risk variables.

**Table 2:** Association between the prevalence of dental caries and socioeconomic risk variables (medical home criteria, household income, and child health insurance) based on the 2016-2019 NSCH data. Statistical tests: ^a^two-sample proportion test; ^b^χ^2^ test FPL, federal poverty level; NSCH, National Survey of Children's Health

Variables	Categories	Possess dental caries, total number (prevalence %)		χ^2^ value	p-Value
		Having tooth decay, n (%)	Not having tooth decay, n (%)		
2016-2017					
Medical home criteria	Met	3053 (8.11)	34615 (91.89)	132.5^a^	<0.001
	Not met	3259 (10.68)	27264 (89.32)		
Household income (FPL)	0-99%	1058 (14.60)	6189 (85.40)	682.70^b^	<0.001
	100-199%	1372 (12.86)	9299 (87.14)		
	200-399%	1985 (9.47)	18984 (90.53)		
	>400%	1900 (6.47)	27448 (93.53)		
Child’s health insurance	Public	1859 (14.69)	10793 (85.31)	806.97^b^	<0.001
	Private	3638 (7.34)	45940 (92.66)		
	Public and private	341 (13.61)	2164 (86.39)		
	Uninsured	365 (14.62)	2131 (85.38)		
2017-2018					
Medical home criteria	Met	2274 (8.47)	24568 (91.53)	23.06^a^	<0.001
	Not met	2591 (11.22)	20501 (88.78)		
Household income (FPL)	0-99%	911 (14.98)	5172 (85.02)	522.37^b^	<0.001
	100-199%	1048 (13.25)	6860 (86.75)		
	200-399%	1520 (9.99)	13690 (90.01)		
	>400%	1388 (6.68)	19378 (93.32)		
Child’s health insurance	Public	1505 (15.16)	8423 (85.84)	555.55^b^	<0.001
	Private	2738 (7.75)	32576 (92.25)		
	Public and private	257 (13.58)	1636 (86.42)		
	Uninsured	284 (13.40)	1835 (86.60)		
2018-2019					
Medical home criteria	Met	2638 (8.55)	28206 (91.45)	159.82^a^	0.001
	Not met	3152 (11.72)	23731 (88.28)		
Household income (FPL)	0-99%	1013 (15.35)	5588 (84.65)	568.23^b^	<0.001
	100-199%	1245 (13.14)	8321 (86.86)		
	200-399%	1895 (10.54)	16085 (89.46)		
	>400%	1640 (6.92)	22072 (93.08)		
Child’s health insurance	Public	1787 (15.48)	9760 (84.52)	625.29^b^	<0.001
	Private	3263 (8.04)	37330 (91.96)		
	Public and private	302 (14.21)	1824 (85.79)		
	Uninsured	343 (12.95)	2306 (87.05)		

**Figure 1:**
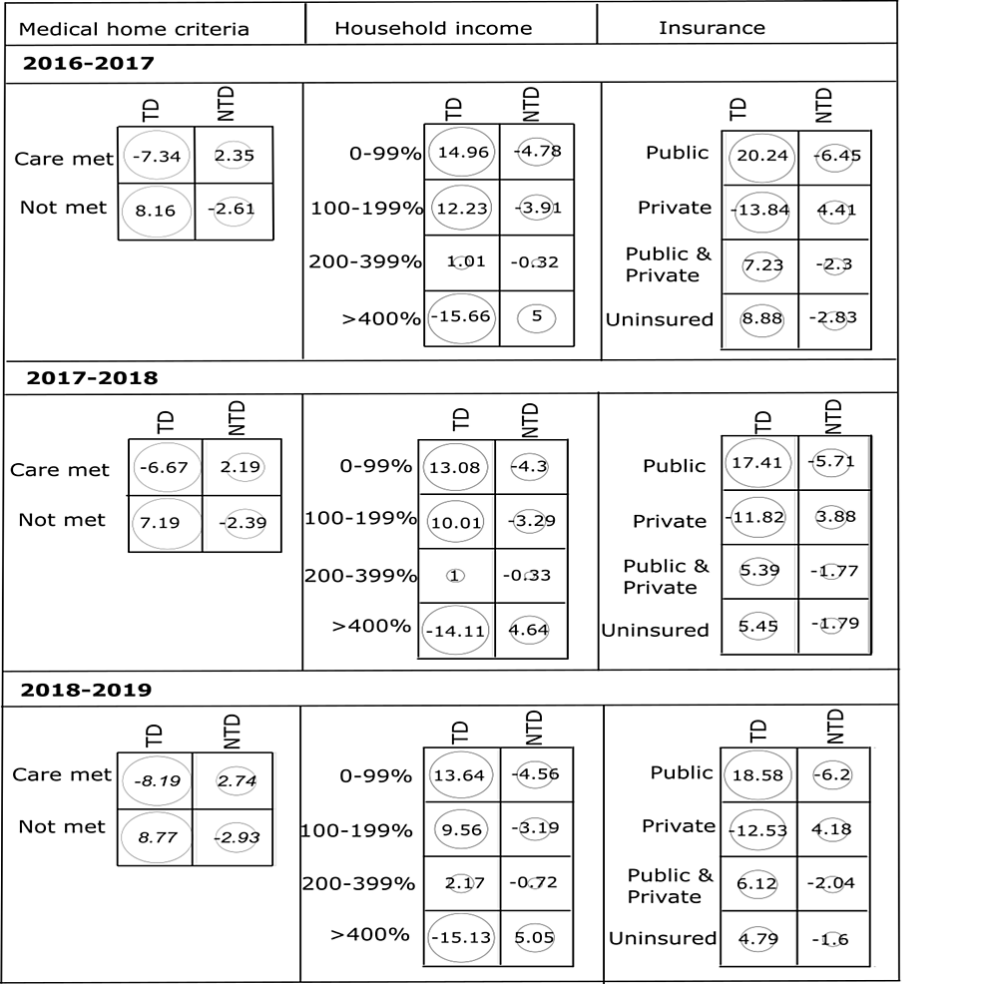
Standardized residuals from χ2 tests for the association between (a) medical home criteria, (b) household income, and (c) insurance of children having decayed teeth (TD) and not having decayed teeth (NTD) during the consecutive three-year survey data from 2016 to 2019.

The prevalence of dental caries among the children who did not meet medical home criteria were 10.68%, 11.22%, and 11.72% in 2016-2017, 2017-2018, 2018-2019, respectively, showing a slightly increasing trend over the years. The prevalence of dental caries was highest among the children from the lowest household income category of 0-99% FPL (14.60-15.35%), while the lowest prevalence of caries was observed in children from the highest household income group of >400% FPL (6.47-6.92%). When considering the insurance coverage type, in 2016-2017 the prevalence of dental caries was comparatively higher for the children with public insurance and uninsured groups, whereas for the next two years the public insurance group had the highest prevalence of dental caries. These preliminary observations of the association of dental caries prevalence with the risk variables were tested for statistical significance. Two-sample proportion tests and χ^2^ tests showed a p-value of <0.001 for all three years, indicating significant statistical associations to exist between prevalence of dental caries and socioeconomic risk factors considered in this study. The test results were further analyzed by calculating the standardized residuals (Figure 1).

Children with dental caries who did not meet medical home criteria, or from low household income (0-99% FPL), or covered by public insurance showed high positive residuals (>7) further confirming a strong positive association of caries with these risk factors. 

To quantify the likelihood that children will have tooth decay when exposed to the respective socioeconomic risk variables, we have also calculated OR (Table 3).

**Table 3:** The odds ratio of having the tooth decay with the exposure of respective socioeconomic risk factors category. FPL, federal poverty level

Exposure group vs other group	2016-2017		2017-2018		2018-2019	
	Odds ratio	p-Value	Odds ratio	p-Value	Odds ratio	p-Value
Medical home criteria						
Care met vs not met	0.74	<0.001	0.73	<0.001	0.70	<0.001
Household income						
0-99% FPL vs 100-199% FPL	1.16	<0.001	1.15	<0.001	1.20	<0.001
0-99% FPL vs 200-399% FPL	1.63	<0.001	1.58	<0.001	1.53	<0.001
0-99% FPL vs >400% FPL	2.47	<0.001	2.45	<0.001	2.43	<0.001
Insurance						
Public vs private	2.17	<0.001	2.12	<0.001	2.09	<0.001
Public vs public and private	1.09	0.159	1.13	0.074	1.10	0.133
Public vs uninsured	1.00	0.932	1.15	0.037	1.23	<0.001

Under the medical home criteria, in 2016-2017 the odds of having tooth decay is approximately 26% lower (OR=0.74; 95% CI=0.70-0.77) if the children met the medical home criteria compared with the children who did not meet the medical home criteria. For the children who met medical home criteria, the OR of having tooth decay became lower over the years (0.74, 0.73, 0.70). In 2016-2017, the ORs under household income indicate that odds of having tooth decay is approximately 16%, 63%, and 147% higher if the children who came from the lowest household income category of 0-99% FPL as opposed to children who came from household income categories of 100-199% FPL, 200-399% FPL, and >400% FPL, respectively. Under the insurance category, the children with public insurance have approximately twice the odds of having tooth decay than children with private insurance in all three years. However, as calculated p-values are greater than 0.001, there is no evidence that the odds of having tooth decay differ among the public group versus private and public group in all three years. Similarly, except for 2018-2019, the ORs are not significant for the public versus uninsured.

Next, we estimated the prevalence of children who received fluoride or other treatment for dental caries in each category of socioeconomic risk variables (Table 4).

**Table 4:** Association between the prevalence of dental treatment and socioeconomic risk variables (medical home criteria, household income, and child health insurance) based on the 2016-2019 NSCH data. Statistical tests: ^a^two-sample proportion test; ^b^χ^2^ test FPL, federal poverty level; NSCH, National Survey of Children's Health

Variables	Categories	Dental treatment, total number (prevalence %)			χ^2^ value	P-Value
		Received fluoride treatment, n (%)	Received treatment without fluoride, n (%)	Not received treatment, n (%)		
2016-2017						
Medical home criteria	Met	21146 (55.94)	11052 (29.24)	5601 (14.82)	11056^a^	<0.001
	Not met	14339 (47.12)	10185 (33.47)	5904 (19.40)		
Household income (FPL)	0-99%	3007 (41.89)	2596 (36.17)	1575 (21.94)	902.42^b^	<0.001
	100-199%	4928 (46.34)	3454 (32.48)	2253 (21.18)		
	200-399%	10798 (51.48)	6391 (30.47)	3786 (18.05)		
	>400%	16755 (56.89)	8803 (29.89)	3893 (13.22)		
Child’s health insurance	Public	5512 (43.74)	4510 (35.79)	2579 (20.47)	1232^b^	<0.001
	Private	27518 (55.34)	14750 (29.66)	7457 (15.00)		
	Public and private	1242 (49.68)	817 (32.68)	441 (17.64)		
	Uninsured	815 (33.04)	798 (32.35)	854 (34.62)		
2017-2018						
Medical home criteria	Met	14950 (55.62)	8042 (29.92)	3888 (14.46)	487.54^a^	<0.001
	Not met	10654 (46.27)	7787 (33.82)	4585 (19.91)		
Household income (FPL)	0-99%	2442 (40.44)	2171 (35.96)	1425 (23.60)	874.36^b^	<0.001
	100-199%	3585 (45.42)	2608 (33.04)	1700 (21.54)		
	200-399%	7781 (51.21)	4659 (30.66)	2755 (18.13)		
	>400%	11799 (56.75)	6396 (30.76)	8475 (12.48)		
Child’s health insurance	Public	4278 (43.27)	3534 (35.75)	2074 (20.98)	996.57^b^	<0.001
	Private	19417 (54.91)	10741 (30.37)	5206 (14.72)		
	Public and private	928 (49.20)	643 (34.09)	315 (16.70)		
	Uninsured	726 (34.59)	643 (30.63)	730 (34.78)		
2018-2019						
Medical home criteria	Met	17256 (56.07)	9275 (30.14)	4243 (13.79)	637.69^a^	<0.001
	Not met	12370 (46.29)	9079 (33.97)	5275 (19.74)		
Household income (FPL)	0-99%	2512 (38.47)	2427 (37.17)	1590 (24.35)	1240.7^b^	<0.001
	100-199%	4250 (45.12)	3144 (33.38)	2026 (21.51)		
	200-399%	9279 (51.79)	5527 (30.85)	3112 (17.37)		
	>400%	13587 (57.47)	7262 (30.72)	2793 (11.81)		
Child’s health insurance	Public	4931 (43.04)	4124 (35.99)	2403 (20.97)	1416.7^b^	<0.001
	Private	22470 (55.47)	12370 (30.53)	5672 (14.00)		
	Public and private	1052 (49.67)	733 (34.61)	333 (15.72)		
	Uninsured	870 (33.23)	815 (31.13)	933 (35.64)		

**Figure 2:**
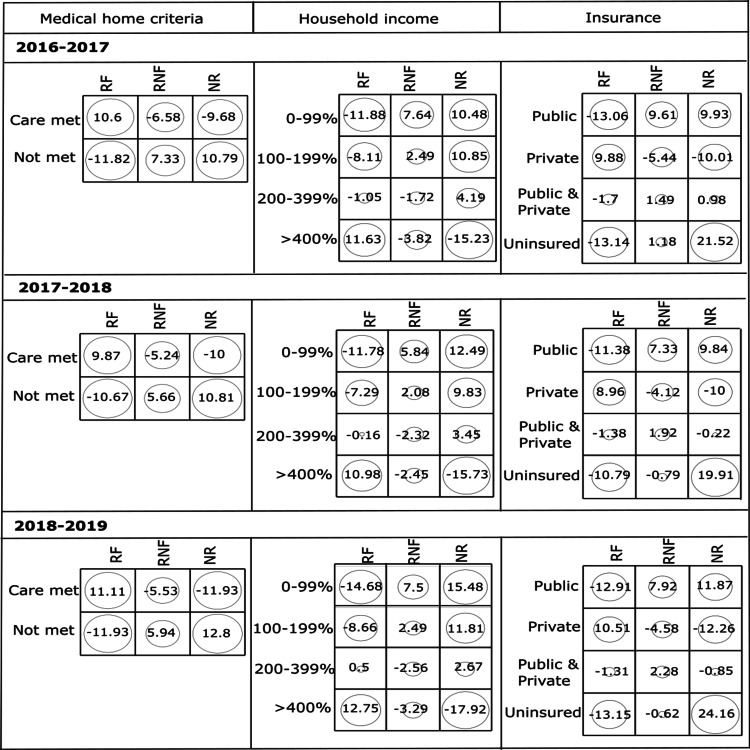
Standardized residuals from χ2 tests for the association between (a) medical home criteria, (b) household income, and (c) insurance of children who received fluoride treatment (RF), received treatment without fluoride (RNF), and did not receive treatment (NR) during the consecutive three-year survey data from 2016 to 2019.

The prevalence of dental treatment without fluoride and untreated caries were highest among children who did not meet medical home criteria. The children from the lowest household income category (0-99% FPL) also showed the highest prevalence of untreated caries and dental treatment without fluoride. When considering insurance status, we observed that children with public insurance have the highest prevalence of dental treatment without fluoride whereas uninsured children have the highest prevalence of untreated caries. The χ^2^ test of independence showed a p-value of <0.001, implying a significant association between socioeconomic risk factors and the prevalence of dental fluoride treatment. Figure 2 shows that the RF group and the children who met the medical home criteria have strong positive association with high positive residuals whereas both RNF and NR groups are positively associated with the children who did not meet the medical home criteria in all three years.

When household income is considered, there is a strong positive association among RF groups with a high household income (>400% FPL). In 2016-2017, the children from the lowest household income (0-99% FPL) were more associated with the RNF group, meanwhile the children from low household income categories of 0-99% FPL and 100%-199% FPL were associated with the NR group. In the other two years (2017-2018, 2018-2019), it is evident that the children from the lowest household income (0-99% FPL) were strongly associated with both RNF and NR groups. When considering insurance, children having private insurance, public insurance, and uninsured became strongly associated with the RF, RNF, and NR groups, respectively, in all three years.

The ORs were followed by the residual analysis in order to quantify the influence of each category on the highly associated category with not receiving dental treatment under each socioeconomic risk factor. To this effect, care met, 0-99% FPL, and uninsured were selected as the reference categories (exposure group) for medical home criteria, household income, and insurance, respectively. The ORs presented in Table 5 show that under medical home criteria, the likelihood of not availing dental treatment is approximately 28%, 32%, and 35% lower for the children who met the medical home criteria than the children who did not meet medical home criteria in consecutive years (2016-2017, 2017-2018, and 2018-2019, respectively).

**Table 5:** The odds ratio of not availing dental treatment with the exposure group of respective socioeconomic risk factors category. FPL, federal poverty level

Exposure group vs other group	2016-2017		2017-2018		2018-2019	
	Odds ratio	p-Value	Odds ratio	p-Value	Odds ratio	p-Value
Medical home criteria						
Care met vs care not met	0.72	<0.001	0.68	<0.001	0.65	<0.001
Household income						
0-99% FPL vs 100-199% FPL	1.04	0.228	1.12	0.004	1.17	<0.001
0-99% FPL vs 200-399% FPL	1.27	<0.001	1.39	<0.001	1.53	<0.001
0-99% FPL vs >400% FPL	1.84	<0.001	2.16	<0.001	2.40	<0.001
Insurance						
Uninsured vs public	2.06	<0.001	2.00	<0.001	2.08	<0.001
Uninsured vs private	3.00	<0.001	3.09	<0.001	3.40	<0.001
Uninsured vs public and private	2.47	<0.001	2.66	<0.001	2.97	<0.001

When considering the household income, it is apparent that the odds of not availing dental treatment is approximately 27%, 39%, and 53% higher if the children hold the lowest household income of 0-99% FPL than the children from the household income of 200-399% FPL in consecutive years (2016-2017, 2017-2018, and 2018-2019, respectively). There is a nearly 100% higher likelihood of not availing dental treatment if the children hold the lowest household income of 0-99% FPL as opposed to the children from the highest household income of >400% FPL. However, the ORs are not significant when comparing the lower income group (0-99% FPL) versus moderate-income group (100-199% FPL) except for the year 2018-2019. Furthermore, it is evident that the likelihood of not availing dental treatment is nearly two, 2.5, and three times higher if the children are uninsured as opposed to children having the insurance as public, a combination of public and private insurance, and private, respectively.

## Discussion

Sociodemographic factors such as age, race, and gender of the children have shown to be established risk factors that impact the prevalence of dental caries and utilization of dental services [21]. We wanted to determine the extent to which the selected socioeconomic factors would affect the same for children in the USA.

The first concept of a medical home was published by the American Academy of Pediatrics (AAP) in 1992 with an updated policy statement in 2002. Accessible, family-centered, continuous, comprehensive, coordinated, compassionate, and culturally effective care are the desirable features of a medical home. The medical home translates to a place where the child has one personal doctor or nurse meeting the well and sick needs of the child and placing referrals as needed, thus rendering family-centered coordinated care [22,23]. Early interventions to obtain good oral health are cost-effective and give the child a good quality of life by preventing ECC. Early dental care can also help pick up other dental abnormalities with a timely workup and interventions. Physician shortage is a nationwide issue, which includes pediatricians [24]. According to the Health Resources and Services Administration (HRSA) estimates in 2018, a shortage of 10,802 dentists in the USA was noted with a projection of increase of dentists through 2037 [25]. Pediatric dentists (PDs) are in shorter supply than general dentists (GDs) [26]. PDs are better trained than the GD to tackle child oral health issues and children behavioral trends around dental care. According to one study, children seen by a PD were 51% more likely to have received fluoride treatment than children seen by a GD [27]. In another recent study, it was noted that younger children with an established medical home had a higher likelihood of receiving preventative dental care [28]. Availability and supply of dentists both play a key role in getting a child established with one. In another study, it was noted that compared to adolescence, early childhood was more sensitive to dentist supply [29]. As noted in previous literature, our study also shows that children who have not met the criteria for a medical home had the higher percentage, associative risk, and odds of having dental caries and not availing dental treatment compared to those who met the criteria for a medical home across all three study years.

Dental visits since 1997 to 2018 have increased over the years across household income of children aged 2-17 years [30]. A study in preschool-aged children shows that income of the family is inversely associated with ECC [31]. Children from low-income families face higher levels of dental disease and have lower frequency of using dental services. Some of the other factors that may provide a hindrance to access dental care for children for low-income families are being able to take off work for a dental appointment, travel arrangements to get to the appointment in places such as rural areas, and arranging for child care [32]. In a recent study, children aged two to five years from lower income and education households were found to have lesser likelihood of receiving preventative dental care [28]. The uninsured rate for children in poverty increased by 1.6% points from 2018 to 2020. Overall, 77.4% of children in poverty were covered by public health insurance [33]. In our study, the highest percentage, associative risk and increased odds for dental caries, and not availing dental treatment were found to be in the children from lower household income (0-99% FPL group) across all three study years compared to the other household income groups.

Having insurance has been positively correlated with availing dental services among children [2]. Around 4.3 million children under 19 years of age (5.6% of all children) were reported to be without health coverage for a calendar year in 2020 [33]. However, there has been a 15.4% rise in children covered by public health insurance from 2005 to 2016, essentially narrowing down the gap between public and private insurance dental utilization [34]. There is a low participation of GDs in treating children with public health insurance, and in one study the reimbursement rates and patient compliance with appointments seemed to be important determinants [35]. In another study, it has been found that in children less than six years of age, establishing a dental home by one year of age could help reduce disease burden [36]. According to our analysis, the public insurance group had the higher percentage, associative risk, and odds of dental caries across all three study years compared to the other insurance groups. The highest percentage, associative risk, and odds of not availing dental treatment were seen in the uninsured group across these time periods under study.

Limitations

The cross-sectional survey data used might have been subject to recall bias by the respondents. Details on the types of nonfluoride treatment the children received were not available in the database.

## Conclusions

Our study findings reveal that certain groups of children under each socioeconomic risk factor such as those who did not meet medical home criteria, those from the lowest household income, public insured, and uninsured are more at risk of developing dental caries and not receiving dental treatment. As a consequence, the study implies that increased support and expansion of public health insurance will benefit oral health care for the children. Pediatricians play a key role in helping initiate the establishment of a dental home for the children. During well-child visits, inquiring to see if the child has a dentist and providing the families with dental referrals will help establish one. In the interim, fluoride varnish application by the pediatrician per existing guidelines will help the child get preventative dental care.
